# Editorial: Insights in Aging Psychiatry: 2021

**DOI:** 10.3389/fpsyt.2022.901931

**Published:** 2022-04-15

**Authors:** Gianfranco Spalletta, Nerisa Banaj, Federica Piras

**Affiliations:** Laboratory of Neuropsychiatry, Santa Lucia Foudation IRCCS, Rome, Italy

**Keywords:** aging, psychiatry, editorial, caregivers, minorities

## Introduction

Over the coming decades, the worldwide population aged 65 and older is projected to triple to 1.5 billion by mid-century (1). The continuously growing share of older person in the population rose concerns regarding seniors' mental healthcare as one out of five individuals aged 60 years and above reported having mental or neurological disorders, and these accounted for 6.6% of all disability adjusted life years (DALYs) (1, 2). Given the burden that mental illnesses pose on healthcare, social security systems and economy, also severely affecting individuals' wellbeing, the present Research Topic was launched to shed light on novel developments, and to inspire further interest and new research efforts in the field of Aging Psychiatry. Major challenges in this sector are represented by the need to identify the multiple risk and vulnerability factors contributing to the onset of mental disorders in seniors (potentially taking advantage of new data mining techniques that will overcome the limitations of traditional explanatory statistical analysis models), and the urgency to guarantee a successful and dignified aging for persons with mental health conditions (especially when they belong to minority groups). An additional demanding task is to establish definite guidelines for assessment and treatment, also considering the opportunity offered by the global diffusion of technology.

Findings and perspectives from contributors of this Research Topic highlight the need to promote a person-centered mental healthcare in the elderlies, considering their multiple and interdependent vulnerabilities, which often derive from long-lasting stressful conditions due to the intersection of ageism and other contextual factors (poverty, sexual discrimination, social isolation for the present pandemic, etc.).

## Stressors and Risk Factors Increasing Seniors' Vulnerability to Mental Disorders

Given the need for screening high-risk groups to prevent the onset of the most frequent mental illness in the elderlies (i.e., depression) several studies tried to identify vulnerability factors using different techniques and algorithms. By developing a predictive model for depressive disorders using stacking ensemble and naive Bayesian nomogram, Byeon et al. evidenced the necessity to continuously evaluate multiple risk factors (i.e., stress perception, subjective health, consumption of polyunsaturated fatty acids, mean daily sitting hours, and mean daily sleeping hours) together with various measurable factors such as social support. The resulting graphical calculating device (the nomogram) can be used in primary care to visually and easily identify groups of seniors at higher risk for depressive disorders and to monitor multiple (and probably not independent) risk factors. Since symptoms of senile depression in the early stage are mild (and seniors usually visit the doctor's office only in case of severe symptomatology) the presented innovative method will ease the process of detecting and managing depressive disorders in older people living alone since the first manifestation of symptoms, thus reducing the risk of developing cognitive impairment that is usually observed in late life depression.

Indeed, Zhang M. et al. by exploring the effect of neuropsychiatric symptoms on cognitive impairments in patients with late-life depression (LLD) demonstrated that emotional, behavioral, and psychotic manifestations impacted cognition. This confirms that behavioral and psychiatric symptoms should be comprehensively evaluated in clinical practice, not only as a potential predictive marker for cognitive decline but also for their significant effect on prognosis (3). The results suggest that early evaluation and interventions for psychiatric symptoms, especially behavioral manifestations during periods of recovery, are of great significance for improving the long-term prognosis of LLD patients.

The loss of physiologic reserve, which manifests in seniors as exhaustion, weakness, low physical activity, slowness, and weight loss (with one/two symptoms characterizing the pre-frailty geriatric syndrome and three or more the frailty state) is another risk factor for the onset of a mental disorder, particularly depression. Zhang L. et al. actually demonstrated, in a big longitudinal cohort of retired seniors in China, that the slowness, weakness and exhaustion components of both syndromes were associated, in the short and long term, with an increased risk of depressive symptoms when other additional risk factors (socio-demographic characteristics, health conditions, and status) were removed. Since slowness, weakness and exhaustion are early markers of depression, and share some risk and pathogenic factors with mood disorders (e.g., the presence of low grade inflammation) interventions designed to prevent the onset of depressive symptoms are very useful. They may, in the long-term, reduce the frail syndrome occurrence in middle-aged and older adults and block the cycle of frailty leading to disability, diseases, physical and cognitive impairments, psychosocial risk factors, and geriatric syndromes (e.g., falls, delirium, and urinary incontinence) (4).

Long-lasting stressful conditions may cause exhaustion of resilience factors and increased depression also in formal and informal caregivers of patients with dementia as demonstrated by Bussè et al. In their paper, they showed that self-perceived psychological stress-related symptoms were significantly increased after 1 year of COVID-19 pandemic compared to baseline measurements. Notably, depression was the most frequently reported symptom 1 year after the pandemic onset, followed by irritability, anxiety, and sleep disturbances. Increased levels of depressive symptoms were predicted by female gender, lower education, perceived isolation, and overwhelm at the onset of the pandemic. Since restrictions imposed by the SARS-CoV-2 coronavirus pandemic led to a significant disruption of health and formal care services (5, 6), an additional burden was posed on informal caregivers, particularly those of patients with dementia and other neurocognitive disorders. Support interventions targeting multiple levels of the stress/health model could produce a significant improvement in caregivers' wellbeing, while psychoeducational interventions may relieve anxiety, enhance awareness and healthy behaviors and reduce family conflicts.

Genetic heritage (particularly the presence of mutations associated with a progressive neurodegenerative disease such as Huntington's disease) and defined personality profiles may be additional risk factors for the onset of psychiatric disorders in the long-term. Moschini et al. showed that the number (within the non-pathological range) of trinucleotide repeats in the Huntingtin gene (the DNA mutation responsible for Huntington's disease) is associated with definite personality traits in people experiencing a subjective decline in cognitive function who progressed to a Mild Cognitive Impairment, but not in those who remained stable. In particular, lower levels of conscientiousness and energy, and higher levels of emotional stability were correlated with higher number of repeats, and both associated with progression of cognitive decline and neuropathological findings consistent with Alzheimer's disease. These associations were independent from possible confounding variables and were not influenced by the presence of depressive symptoms, suggesting that the repeat length below the pathological threshold does not affect mood, although it may mediate the effect of personality traits on neurodegeneration and progression of cognitive decline.

Finally, belonging to gender and or sexual minority groups could be a further stressor negatively impacting on the health, wellbeing, and successful aging outcomes of older people. For example, the growing population of individuals who identify as lesbian, gay bisexual, transgender, queer, intersex, asexual and other (LGBTQIA+), are more exposed to stress factors due to the intersections of ageism, homophobia, biphobia, transphobia, racism, or poverty (7). In their opinion article, Pereira and Banerjee argument that older LGBTQIA+ people are subject to unique stressors associated with their minority status, and may face double discrimination due to their age and their LGBTQIA+ identity, making them more likely to experience health disparities. Older LGBTQIA+ individuals represent a diverse group of people who are still exposed to adversity, stigma, marginalization, and discrimination, with a greater probability of isolation, less social support, and therefore more at risk for having worse physical, mental, and social health indicators. Heteronormative aging models do not adapt to the specific needs of older LGBTQIA+ people and are marked by a double stigmatization lens (LGBTQIA+-phobia and ageism).

## Tailored Approaches to Psychiatric Disorders in Old Age

In a comprehensive perspective paper, Banerjee et al. discuss the importance of the multi-dimensional framework of dignity as the anchor to a person-centered mental healthcare for the elderlies. They highlight the various components of dignity in older people, the impact of ageism and mental health interventions based on rights, respect, and equality (including dignity therapy) for older adults and encourage an urgent call for action for a legally binding United Nations convention on the human rights in this population.

One of the aspects considered in the previous perspective paper, i.e., the need to protect people with impaired decision-making ability, in the respect of the person's dignity, was the focus of the study by Wied et al. in which the systematic development and implementation of support tools to enhance informed consent processes (the so-called enhanced consent procedures/ECP) for lumbar puncture treatment in persons with dementia is described. Eight possible tools were developed by involving an interdisciplinary transnational expert group (including a standardized interview, lists of keywords, priority cards, etc.) which should be proposed to patients by facilitators with a person-centered attitude. Authors conclude that the proposed tools should be selected (and not administered as a whole in their standard form) according to patients' individual needs and resources and to abilities (e.g., qualifications) of the practitioner. Their findings can serve as a selection of possibilities to support patients with dementia in decision-making and might help practitioners achieving an appropriate balance between the autonomy and protection of patients in complex decision-making.

Indeed, treatment strategies (as well as the perception of neuropsychiatric symptoms) in dementia care centers are not standardized (at least in Italy) as highlighted by a multicenter national survey performed through a semi-structured interview by D'Antonio et al. Results showed that the perceived frequency of neuropsychiatric symptoms was 74% and they were detected by means of a clinical assessment for 96.3% or a caregiver interview for 97%. The survey also revealed differences in symptoms perception, treatment options (where most of the centers apply non-pharmacological treatments) and observed side effect according to the clinical setting. The authors explained such variability by the absence of clear guidelines, by differences in patients' characteristics and by clinical practice based on subjective experience, highlighting the need of guidelines for the pharmacological treatment of neuropsychiatric symptoms in dementia.

New treatment strategies may include individualized interventions as reported by Ishimaru et al. who developed a new assessment tool (the Photo Assessment of Living Environment -PA-LE-) to understand the environmental context of delusion of theft in dementia, the most prevalent form of delusion in the elderlies suffering from cognitive decline. Familial interviews were conducted to assess the phenomenological features of the disorder and non-pharmacological approaches were tailored to the patients' environmental and psychological states, referring to the interview and the proposed innovative tool PA-LE. This included environmental adjustment or increasing self-esteem. Antipsychotics were also prescribed. Environmental and psychological triggers of delusion were improved by the interventions, and the patients had uneventful courses without active delusions. Authors concluded that evaluating patients' homes using photos could detect the environmental context of delusion of theft among patients with Alzheimer's dementia and assist in case management.

Additionally, some relief from psychiatric symptoms (particularly depression) may come from the opportunity offered by the global diffusion of technology, as demonstrated by Yang et al. who investigated the effect of internet use on depressive symptoms on 7,801 adults over 60 from the 2018 China Family Panel Studies. Results showed that older adults who used the internet reported lower depression scores particularly in those with higher frequency of use and especially in the younger group and in females. Moreover, using the Internet for social contact and entertainment decreased depression scores, but when the Internet was used for learning, working, and for commercial activities, the relief of depressive symptoms disappeared. This would suggest that Internet usage determined some relief from depression by increasing the frequency of contact with children and life enjoyment. Thus, policies should be designed to ensure that all ages have easy access to the Internet.

## Conclusion

Overall, contributions of the present Research Topic covered a wide variety of issues related to the psychiatry of aging, ranging from identifying neuropsychiatric symptoms, their impact not only on patients but also on caregivers, and to physicians' perceptions and treatment strategies. Furthermore, particular attention was paid to individual needs and the right to make conscious decisions for healthcare, highlighting the fact that the elderlies, and particularly those from minority groups, should be considered in their complexity and the scientific world should promote new actions in this direction.

## Author Contributions

GS, NB, and FP contributed to conception and design of the paper. NB and FP wrote the first draft of the manuscript. GS supervised the manuscript. All authors contributed to manuscript revision, read, and approved the submitted version.

## Funding

This paper was supported by the Italian Ministry of Health Grants RC 2020-21-22/A.

## Conflict of Interest

The authors declare that the research was conducted in the absence of any commercial or financial relationships that could be construed as a potential conflict of interest.

## Publisher's Note

All claims expressed in this article are solely those of the authors and do not necessarily represent those of their affiliated organizations, or those of the publisher, the editors and the reviewers. Any product that may be evaluated in this article, or claim that may be made by its manufacturer, is not guaranteed or endorsed by the publisher.
